# Efficacy of pulsed radiofrequency stimulation in patients with chronic pain: a narrative review

**DOI:** 10.3389/fpain.2025.1544909

**Published:** 2025-08-12

**Authors:** Wei Lin, Lingling Lou, Dawei Chu, Yidong Lv, Liujun Tian, Bin Wang

**Affiliations:** ^1^ Department of Pain, Weifang People’s Hospital, Weifang, Shandong Province, China; ^2^ College of Clinical Medicine, Shandong Second Medical University, Weifang, Shandong Province, China; ^3^ Department of Neurology II, Affiliated Hospital of Shandong Second Medical University, Weifang, Shandong Province, China

**Keywords:** pulsed radiofrequency, chronic pain, mechanism of action, clinical citation, efficacy assessment

## Abstract

**Objectives:**

The review aimed to evaluate the efficacy of pulsed radiofrequency (PRF) in treating chronic pain by analyzing recent literature.

**Study design:**

This is a narrative review of relevant articles on the effectiveness of PRF for chronic pain.

**Methods:**

Search for papers published between November 2014 and November 2024 in the PubMed database that use PRF to treat chronic pain. We used “Pulsed radiofrequency, PRF, Pulsed RF for Pain, chronic pain, neuropathic pain, cancer pain, and osteoarthritis pain” as search terms. Inclusion criteria are as follows: (1) Patients are clearly diagnosed with chronic pain according to the standards of the International Association for the Study of Pain; (2) Pulsed radiofrequency is used to treat chronic pain; (3) Follow-up assessments are conducted to evaluate the degree of pain relief after PRF treatment; (4) Review articles and articles not related to the treatment of chronic pain are excluded.

**Results:**

Preliminary searches yielded 368 relevant articles. After reviewing the titles and abstracts and evaluating the full texts, we ultimately included 80 articles. These articles cover research on pulsed radiofrequency treatment for various chronic pain conditions, including neuropathic pain, osteoarthritis pain, and cancer pain. The study types are diverse, including randomized controlled trials, cohort studies, and case reports. The publication dates of the articles range from 2014 to 2024, ensuring the timeliness and comprehensiveness of the research findings, which reflect the latest advancements and outcomes in the field of pulsed radiofrequency treatment for chronic pain.

**Limitations:**

This review did not include studies indexed in databases other than PubMed.

**Conclusion:**

This article reviews the research progress of pulsed radiofrequency technology in the field of chronic pain treatment. By searching and analyzing relevant literature from recent years, it summarizes the research findings on the mechanisms of PRF in treating chronic pain, its clinical applications, efficacy evaluation, and safety, and discusses future research directions. This is helpful for clinical physicians to develop more scientific treatment plans when managing chronic pain patients.

## Introduction

Chronic pain severely affects the quality of life of patients, imposing a heavy physical and mental burden, and presenting significant challenges for clinical treatment. Pulsed Radiofrequency (PRF) therapy, as an emerging technology, is increasingly being applied to alleviate various types of pain, including neuropathic pain, joint pain, back pain, shoulder pain, and cancer-related pain (1–5). This technique is an improved form of traditional continuous radiofrequency (RF), which was accidentally proposed in 1993. The first successful PRF procedure for the lumbar dorsal root ganglion was performed on February 1, 1996. The core of the technique lies in using intermittent radiofrequency currents to achieve therapeutic goals.

Compared to traditional RF thermocoagulation techniques, PRF offers significant advantages such as ease of operation, high safety, minimal trauma, and fewer complications. Traditional RF technology uses high temperatures (>60°C) to cause protein coagulation and denaturation in tissues, effectively blocking pain signal transmission (6–13). However, it often results in post-operative complications such as nerve damage, numbness, muscle atrophy, itching, and recurrence of pain, with recurrence rates increasing over time. In contrast, PRF achieves technological innovation through parameter optimization: the RF device emits high-frequency alternating current at 500 kHz with a 2 Hz pulse frequency, with each pulse lasting 20 ms and a 480 ms interval between pulses. This scientific combination of “pulse duration + interval time” allows heat around the nerve tissue to dissipate adequately, ensuring that the temperature at the electrode tip remains below 42°C, far below the protein denaturation threshold, thus preventing irreversible tissue damage (14–16).

Regarding the mechanism of action, the 42°C temperature threshold has dual clinical significance: it modulates nerve excitability through the electric field effect (such as promoting c-Fos expression and inhibiting the release of pain neurotransmitters) and helps avoid the risk of thermal damage. The synergistic effect of the 500 kHz RF frequency and 2 Hz pulse frequency is key to achieving “nerve modulation rather than destruction.” The 500 kHz frequency determines the depth of the electric field penetration and the amplitude of the change in nerve cell membrane potential, while the 2 Hz pulse frequency controls the periodicity of nerve stimulation. Together, they induce periodic opening of the ion channels in the cell membrane, modulating pain signal transmission while effectively avoiding the heat accumulation effect caused by continuous high-frequency current. This precise parameter design ensures that PRF offers both therapeutic efficacy and tissue safety in clinical applications (17, 18).

Here, the literature was reviewed to establish the effectiveness of pulsed radiofrequency treatment for various chronic pain conditions.

## Methods

Searched for papers published between November 2014 and November 2024 on the use of PRF to treat chronic pain in PubMed. We used PRF or Pulse Repetition Frequency, pain or (chronic pain, neuropathic pain, cancer pain, and osteoarthritis pain) as search terms. During the data screening process, we first conducted a preliminary screening based on the titles and abstracts of the literature, eliminating obviously irrelevant documents, such as those whose research content is unrelated to pulsed radiofrequency treatment of chronic pain or whose subjects are not chronic pain patients. For the remaining literature after the initial screening, the full text is further read, and strict screening is conducted based on the pre-established inclusion and exclusion criteria. Inclusion criteria include: (1) the study type is clinical research; (2) the study subjects are clearly defined as chronic pain patients (such as chronic headache, chronic neuropathic pain, chronic joint pain, etc.); (3) the use of pulsed radiofrequency treatment methods; (4) clear efficacy evaluation indicators. Exclusion criteria include: (1) literature that has been published multiple times; (2) literature for which the full text cannot be accessed or key information is missing; (3) literature with excessively low research quality, such as small sample sizes or unreasonable study designs. Through this screening process, we ensure that the literature included in the final review has high quality and relevance, thereby providing a reliable data foundation for accurately analyzing the efficacy of pulsed radiofrequency treatment for chronic pain.

## Results

**Figure 1:**
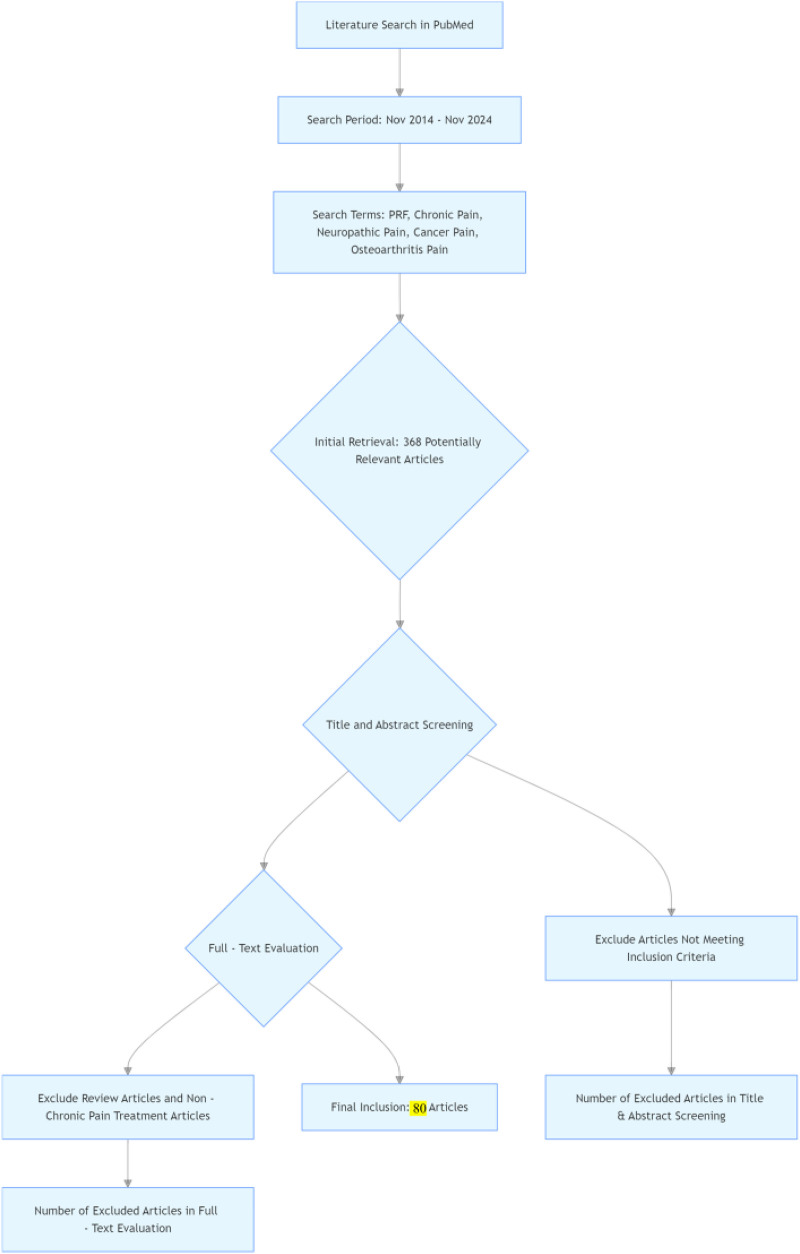
Flowchart of the research method.

In the initial literature search, a total of 368 potentially relevant articles were identified. After reviewing the titles and abstracts and conducting a comprehensive evaluation of the articles based on the full-text, 80 publications were finally included in this review. Among the included studies, 12 studies (6–10, 19–25) and 18 studies (3, 26–42) applied PRF for the treatment of neck and back pain, respectively, and 6 studies (1, 43–47) investigated the use of PRF for the treatment of chronic neuralgia, seven studies (11–13, 48–51) for headache, eight studies (4, 52–57) for shoulder pain, 18 studies (2, 58–74) for arthralgia, and 10 studies (5, 75–83) for other pain (Figure 1).

## Discussion

### Chronic cervical spine radiculopathy

Cervical Spine Radiculopathy is defined as radiating pain felt in the upper extremity, caused by irritation or compression of the cervical spine, nerve roots, or both, with C7 and C6 being the most commonly affected ganglia (84). A systematic review on Cervical Spine Radiculopathy showed an incidence and prevalence range of 1.21–5.8/1,000 people (85).

Indeed, numerous studies have been devoted to validating the efficacy and safety of pulsed radiofrequency (PRF) therapy in relieving chronic Cervical Spine Radiculopathy. For patients with chronic Cervical Spine Radiculopathy, we carefully screened and identified 13 relevant studies (3, 26–42), which invariably confirmed the significant efficacy of PRF treatment.

Of these 13 studies, five were rigorous randomized controlled trials (RCTs) (20–23, 25), of which the study conducted by Gokhan Yildiz et al. (23) was particularly notable. By comparing the therapeutic effects of selective nerve root pulsed radiofrequency (ULSD-SNRPRF) with paracentral interlaminar epidural steroid injection (FL-CIESI), they found that the NRS scores of the patients in both groups decreased significantly after the treatment, and the difference did not reach the level of statistical significance, which implied that both methods were effective in relieving pain. In addition, an experiment used bipolar radiofrequency pulsed technology to treat patients with chronic neck pain, and the results showed that 50% of the patients in the PRF treatment group had a postoperative pain reduction of 50% or more, and this effect lasted for about 3 months (6).

In 2016, Wang et al. (20) conducted a randomized controlled trial, which first confirmed that cervical nerve block combined with pulsed radiofrequency (PRF) treatment was significantly more effective than PRF alone for chronic cervicogenic pain. This study not only established the effectiveness of PRF in treating chronic cervicogenic pain, but also proposed a synergistic treatment strategy combining nerve block or nerve blockade with PRF, providing important clinical evidence for practice.

In 2021, another study involving 42 patients with chronic cervical radicular pain (10) further explored the long-term benefits of PRF. The results showed that compared to steroid injections alone, the combination of PRF and cervical nerve block significantly reduced the pain scores of patients at 3 months post-treatment, with the effect lasting up to 6 months. Additionally, the neck disability index (NDI) of patients in the combined treatment group showed continuous improvement at both 3 and 6 months post-treatment, further demonstrating the long-term effect of PRF in promoting functional recovery. This study provided more detailed data on the therapeutic time window for PRF in chronic cervicogenic pain.

Furthermore, seven additional studies [including prospective studies (9, 10), cohort analyses (6), and case reports (7, 8)] have further validated the efficacy and safety of PRF. Notably, one case report (8) involved a patient with chronic pain due to cervical disc disease (NRS score of 7/10). After receiving intervertebral PRF treatment, the pain score decreased to 0 within 2 weeks and only showed slight rebound (score of 2) at 3-month follow-up. No adverse reactions were reported throughout the treatment process, providing strong evidence for the safety and rapid onset of PRF.

In conclusion, existing evidence suggests that PRF may alleviate pain through various mechanisms, primarily by modulating nerve conduction, reducing inflammation, and promoting nerve repair.

### Chronic neuralgia

When pain originates from lesions and diseases of the central or peripheral somatosensory nervous system, we call it neuropathic pain. The scope of chronic neuropathic pain is broad, encompassing a wide range of types including painful peripheral polyneuropathy, postherpetic neuralgia, pain caused by traumatic nerve injury, and pain caused by damage to the spinal cord or brain (86, 87). To date, treatments for neuralgia have mainly included nerve tissue therapy and medication, however, no single therapy has yet to be hailed as the best choice for neuralgia treatment. Recently, pulsed radiofrequency (PRF) technology has emerged in the field of neuralgia as a safe and potentially effective treatment.

Indeed, numerous studies are actively exploring the efficacy and safety of pulsed radiofrequency (PRF) technology in the management of neuropathic pain. To date, we have screened 6 relevant studies (1, 43–47) for in-depth analysis. Among these 6 studies, 2 were rigorous randomized controlled trials (RCTs) (43, 44), providing strong evidence for the therapeutic efficacy of PRF.

In 2016, Dan Li et al. (43) conducted a study comparing the efficacy of three treatment regimens: radiofrequency pulsed therapy, nerve block, and pulsed radiofrequency combined with nerve block. The results of their study showed that visual analog scores (VAS) decreased in all patients after treatment, with the most significant decrease in VAS scores in the pulsed radiofrequency combined nerve block group, and did not show a significant difference in the incidence of adverse effects among the four groups.

While in 2022, Shao-jun Li et al. (44) analyzed the effect of PRF in the treatment of postherpetic neuralgia from the perspective of different needle tip positions. They found that when the needle tip was positioned in the area between the medial and lateral edges of the adjacent pedicle root, it was able to significantly relieve patients' pain and effectively improve their quality of life. These two randomized controlled trials not only validated the effectiveness of PRF in the treatment of neuralgia, but also further revealed that selective neurotomy or nerve block in combination with PRF can further enhance the treatment effect.

The other four studies—containing two retrospective analyses along with two in-depth case studies—provide equally strong evidence of the remarkable effectiveness of PRF in the treatment of neuralgia. Of particular note, one of these studies focused on 20 patients with pubic neuralgia who underwent neuropulsed radiofrequency treatment. The results showed that 79% of the patients achieved a Patient General Impression of Improvement scale (PGI-I) score of 1 or 2 at 3 months post-treatment, and the efficacy was long-lasting, spanning 2.3 to 8.8 years. Of note, only 1 patient (5.3%) experienced an increase in pain after PRF treatment. During follow-up, patients reported only transient vaginal bleeding, and none of the patients who participated in the study experienced infections, neurologic complications, or incontinence.

These studies also suggest that PRF may be effective in relieving chronic neuropathic pain symptoms through mechanisms such as modulating the excitability of damaged nerves, attenuating the neuroinflammatory response and promoting nerve regeneration.

### Chronic joint pain

Individual discomfort and consequent pain caused by systemic joint hyperactivity are collectively referred to as chronic widespread pain, also known as joint hyperactivity syndrome (88). In recent years, numerous studies have been devoted to validating the efficacy and safety of pulsed radiofrequency (PRF) for the treatment of chronic joint pain. Eighteen relevant studies have been identified for patients with chronic joint pain (3, 26–42), all of which have consistently confirmed the significant efficacy of PRF treatment.

Of these 18 studies, 9 were rigorously designed randomized controlled trials (RCTs) (2, 58, 60, 62, 66, 69, 70, 73, 74). Of these, the study conducted by Qi Han et al. (58) was particularly notable, in which they compared the effects of PRF treatment alone with PRF combined with physical therapy (PS). The results of the study showed that 77.4% of patients in the PRF combined with PS group reported more than 20% improvement in their PT 60 degrees/second postoperatively, compared to 17.2% in the PS alone group. Similarly, the pulsed radiofrequency combined with PS group excelled in muscle strength improvement at PT 180 degrees/second, with 74.2% of patients showing more than 20% improvement in muscle strength, compared with only 6.9% in the PS alone group. Even more encouragingly, 30 of 31 patients (96.8%) in the PRF combined with PS group reported more than 20% improvement in knee function in their WOMAC scores; in contrast, only 2 of 29 patients (6.9%) in the PS-only group reported similar functional improvement. These randomized controlled trials not only validated the effectiveness of PRF in relieving chronic joint pain, but also revealed that PRF combined with PS or nerve block therapy can further enhance its therapeutic effect.

It is noteworthy that, to date, nine studies have specifically evaluated the therapeutic effects of PRF on chronic knee pain (59, 61–65, 72–74). Of these nine studies, four were randomized controlled trials (58, 62, 70, 71) as well as two others that are not explicitly listed but can be inferred from the context (73, 74)], two were prospective studies (59, 72), and three were retrospective studies (64, 65, 71). Although these studies differed in the specific degree of pain relief, a common thread was that after PRF treatment, patients with chronic knee pain showed significant improvement in their symptoms compared to the pre-treatment period.

By analyzing the above studies, we speculate that PRF may be effective in improving chronic arthralgia by regulating the excitability of intra-articular nerve endings, reducing joint inflammation and promoting cartilage repair.

### Chronic headache

Chronic headache, defined as headache symptoms that last 15 or more days per month and have persisted for at least 3 months, is a key trigger of pain and disability. Specifically, chronic migraine affects approximately 1% to 4% of the population, tension-type headache affects approximately 2.2% of the population, and an even greater 25% to 50% of headache sufferers have their symptoms exacerbated by substance abuse (89).

In recent years, numerous studies have been devoted to exploring the efficacy and safety of pulsed radiofrequency (PRF) for the treatment of headache. Seven relevant studies have been identified for patients with chronic headache (11–13, 48–51), and all of these studies have consistently confirmed the positive effects of PRF treatment.

Of these seven studies, five used a rigorously designed randomized controlled trial (RCT) approach (13, 48–51). Among them, Karaduman Y et al. (49) conducted a study in 2024 comparing the effects of steroid injection with PRF treatment. The results of their study showed that both groups showed improvement in pain compared to the pre-treatment period, and the improvement was more significant in the PRF group, although this difference did not reach the level of statistical significance. On the other hand, Soyoung Kwak et al. (11) analyzed 2 patients with intractable chronic migraine in 2018. These two patients had pre-treatment numeric rating scale (NRS) scores of 8 and 7 out of 10, respectively. Interestingly, the results of the study showed that the headache symptoms of these two patients instead worsened after the first PRF treatment. However, after 2 weeks of treatment, both of their NRS scores decreased to 3 and they did not report any subsequent worsening of pain, with efficacy lasting up to 3 months. In addition, a study by Jun Li et al. (13) in 2020 found that ultrasound-guided pulsed radiofrequency treatment of the C2 nerve significantly improved patients' headache symptoms.

In addition to the 5 RCT studies mentioned above, the remaining 2 studies-including 1 prospective study (48) and 2 case-report studies (11, 12)-have similarly demonstrated the effectiveness of PRF in the treatment of chronic headache.Five other studies, including one prospective study (48), two retrospective studies (13, 50), and two case reports (11, 12), have also demonstrated the effectiveness of PRF in the treatment of chronic headache.

Of particular note, to date, four studies have specifically evaluated the efficacy of PRF in the treatment of chronic migraine (11, 12, 48, 49). Two of these were randomized controlled trials (48, 49) and the other two were case studies (11, 12). Although these studies differed in the specific degree of pain relief, a common thread was that after PRF treatment, chronic migraineurs all showed significant improvement in their symptoms compared to pre-treatment.

Our analysis of the above studies suggests that PRF may be effective in improving chronic migraine symptoms through mechanisms such as regulating the excitability of the trigeminal vascular system, reducing meningeal inflammation, and promoting neuromodulation.

### Other chronic pains

In addition to the aforementioned conditions, pulsed radiofrequency (PRF) technology has been used innovatively in the treatment of chronic pelvic pain (78) and intractable metastatic back pain in the thoracic vertebral body (83).

Chronic pelvic pain syndrome (CPP) is often closely associated with non-pelvic pain conditions, such as fibromyalgia, and non-pain-related complications, such as sleep disorders, emotional issues, and cognitive impairments. These factors intertwine and collectively exacerbate the pain and functional disability in patients. Musculoskeletal pain and dysfunction are commonly observed in CPP patients. While pharmacological and surgical treatments are widely used, the long-term efficacy of these treatments remains difficult to predict. Notably, an interesting study has shown that the combination of upper abdominal lower plexus nerve block and pulsed radiofrequency (PRF) for treating pelvic cancer-related chronic pelvic and perineal pain provides more significant pain relief than the use of upper abdominal lower plexus nerve block alone (5, 76, 78). A vivid case report documents a young, childless, married female patient who sought medical attention for chronic pelvic pain associated with adenomyosis. After various therapies such as oral antispasmodics, non-steroidal anti-inflammatory drugs, birth control pills, gonadotropin-releasing hormone (GnRh) analog hormone therapy, intramuscular diclofenac and laparoscopic adenomyomectomy, she was unable to get rid of the pain (78). However, the introduction of PRF technology brought her a turnaround, with a significant reduction in pain after the treatment and a stable efficacy maintained for five months after several treatments (78).

The intractability of metastatic bone pain, a typical manifestation of cancer pain, is self-evident, as it contains not only injurious pain, but also an admixture of neuropathic pain (90). Although low-dose pregabalin antidepressants in combination with opioids have demonstrated some effectiveness in the treatment of painful bone metastases (91), the pain suffered by patients with bone metastases while moving remains one of the most difficult problems to overcome. In 2015, a study by Young-Chang et al. (83) opened up a new pathway in the treatment of intractable metastatic spinal pain –pulsed radiofrequency therapy of the dorsal root ganglion. The results of the study showed that this technique was able to provide significant pain relief to patients, and the duration of efficacy ranged from 2 to 6 months.

These studies provide strong evidence of the effectiveness of PRF in the treatment of chronic pelvic pain and persistent metastatic back pain in the thoracic spine.

### Mechanisms of PRF for chronic pain

#### Neuromodulatory mechanisms

Partial injury or ligation models of the sciatic nerve are widely used in animal experiments as an important tool for exploring neuropathic pain. Choi et al. (92) delved into the effects of pulsed radiofrequency (PRF) on the rat sciatic nerve at the ultrastructural and biological levels. Compared with continuous radiofrequency (CRF), pulsed radiofrequency-treated sciatic nerves exhibited slight swelling of myelinated axons and had limited ultrastructural effects on collagen-immunized nerve fibers of types I and III, causing only minor damage to myelinated nerve fibers. The analgesic mechanism of PRF lies in its ability to temporarily block nerve signaling and preferentially destroy pain-related sensory fibers (e.g., Aδ and C fibers), with less effect on the larger Aβ nerve fibers responsible for non-pain-related sensory transmission (93).

Boesch et al. (94) compared the effects of CRF and PRF on the saphenous and sciatic nerves, respectively, in Beagles. The results showed that Waller degeneration was observed in saphenous nerves treated with CRF, whereas Waller degeneration was not observed in sciatic nerves treated with PRF. More importantly, neither nerve triggered postoperative pain or motor dysfunction after receiving the corresponding treatment. This finding further confirms the safety and efficacy of combined sciatic and saphenous nerve radiofrequency techniques in the treatment of knee osteoarthritis.

Current research evidence strongly suggests that the neuromodulatory effects of PRF do not cause substantial damage to nerves. On the contrary, pulsed radiofrequency can positively repair damaged nerves by up-regulating the expression of neurotrophic factors and reducing inflammatory responses, and can help to reverse demyelination, demonstrating its great potential in the field of neuroprotection and therapy.

### Inhibits the production of pain substances

Pulsed radiofrequency likewise demonstrated significant analgesic effects at the level of the dorsal horn of the spinal cord. By inhibiting the release of excitatory amino acids (e.g., glutamate, aspartate, and citrulline) induced by nociceptive stimuli in the spinal cord-cerebrospinal fluid, pulsed radiofrequency effectively alleviated the symptoms of neuropathic pain (95). substance P (SP), a neuropeptide released from the central nervous terminals, is directly or indirectly involved in the transmission of nociception through the facilitation of glutamate release at its C-terminus, while the N-terminus mediates a slight analgesic effect with the help of enkephalin (M-ENK). The C-terminus of this neuropeptide, released from the central nervous terminals, is directly or indirectly involved in nociception by facilitating the release of glutamate and other substances, whereas the N-terminus mediates mild analgesic effects with the help of metenkephalin (M-ENK). It is noteworthy that PRF was able to inhibit the expression of SP in the spinal cord of rats with CCI, which in turn elevated the threshold of mechanical foot reduction, demonstrating its analgesic potential (96).

In addition to curbing the production of pain-causing substances, pulsed radiofrequency also exerts analgesic effects by promoting the production of analgesic substances (97). In the central nervous system, the balance between excitatory and inhibitory neurons is critical. Glutamate, the primary excitatory neurotransmitter, works with GABA and glycine, two key inhibitory neurotransmitters, to maintain homeostasis in the system. The strength and polarity of inhibitory neurotransmission in the nervous system are strongly influenced by intracellular chloride ion concentration and potassium-chloride cotransporter protein 2 (KCC2) activity. Pulsed radiofrequency partially restored the function of GABA synapses by augmenting histone acetylation and elevating the expression of KCC2, thereby effectively attenuating the nociceptive sensitization phenomenon (98). In addition, pulsed radiofrequency reinforced noradrenergic and 5-hydroxytryptaminergic downstream pain inhibitory pathways, further exerting its analgesic efficacy (99).

Brain-derived neurotrophic factor (BDNF) and its upstream regulator phosphatidylinositol-3 kinase (PI3K) are also finely regulated by PRF, which reduces the levels of PI3K (100) and p-ERK (101) in the spinal cord by down-regulating the expression of BDNF (100) and insulin-like growth factor 2 (IGF-2) (102), and inhibits the phosphorylation of p-38 and JNK (101, 103) phosphorylation process, thereby altering neuronal plasticity, inhibiting glial cell activation, and significantly improving neuropathic pain triggered by nerve injury. Implementation of PRF treatment to the dorsal root ganglion (DRG) may trigger the adjustment of the neuro-immune axis within the spinal cord, leading to the weakening of the local blood-brain barrier function, which in turn triggers secondary neuroinflammatory changes within the spinal cord (87, 104). In this process, pulsed radiofrequency elevated the pain threshold by inhibiting CCL2 expression and NF-*κ*B phosphorylation (105). At the spinal cord level, pulsed radiofrequency also affects ion channel receptors and effectively curbs the development of neuropathic pain by inhibiting the expression of P2X3 receptors (106) and Cav2.2 protein (107) in the dorsal horn of the spinal cord.

## PRF and electron microscopy

Radiofrequency pulses through the action of radiofrequency currents on biological tissues, lead to damage to cell membranes, degeneration of mitochondria, and changes in other intracellular organelles. They can cause subtle damage to neurons and nerve fibers, particularly alterations in axons, thereby interfering with the transmission of pain signals. Additionally, they may promote a certain degree of tissue repair and regeneration after treatment (108–110). Research has found that electron microscopy, particularly transmission electron microscopy (TEM) and scanning electron microscopy (SEM), provides a visualization tool with nanometer-level resolution for evaluating the biological effects of radiofrequency pulses. This allows for precise observation of changes in cellular ultrastructure, alterations in membrane integrity, and patterns of subcellular organelle damage (109, 110). This multimodal research approach not only provides direct evidence for elucidating the molecular mechanisms of radiofrequency therapy but also enables optimization of treatment parameters through feedback from microscopic structural changes, thus advancing medical development.

### Therapeutic parameters of PRF for chronic pain

After an in-depth analysis of the above pilot study, we found that, after implementing local anesthesia, a 22-gauge radiofrequency puncture needle with a length of 5 mm at the effective end and an overall length of 10 mm (total length of 10 cm and 0.5 cm at the exposed end) was used, and under ultrasound guidance, the needle was accurately inserted along the plane of the puncture up to the ideal stimulation site. Subsequently, the RF therapeutic instrument and electrodes were connected, and the RF electrodes were inserted to start the sensory test. The test parameters were set to a voltage of 0.3 to 0.5 volts and a frequency of 50 Hz. Following the standard pulsed RF mode, the parameters were set to 42°C and 45 volts for 90 s and this was repeated three times. After the procedure, the radiofrequency needle was gently removed, the puncture point was strictly sterilized, and properly dressed and secured with sterile gauze (111, 112). This therapeutic parameter is widely used in practice and has proven its effectiveness.

Treatment duration and cycle time are key factors in the effectiveness of pulsed radiofrequency (PRF). A single PRF treatment is usually short, ranging from a few minutes to a few tens of minutes, but multiple treatments may be required for optimal results. In addition, the treatment cycle needs to be customized to the patient's specific situation to ensure maximum results.

The precise selection of the treatment site and target point has a profound impact on the efficacy of PRF. When determining the treatment site, the root cause of pain and nerve conduction pathways need to be carefully considered. When selecting the target site, the nerve structures associated with pain need to be precisely located to ensure the accuracy and effectiveness of the treatment, thus maximizing the therapeutic effect.

When it comes to the safety of PRF for the treatment of chronic pain, it is highly regarded for its low complication rate and high safety. Numerous studies have shown that patients have not experienced serious complications such as nerve damage, infection or bleeding after PRF treatment. In addition, pulsed radiofrequency treatment has many advantages such as repeatable operation, no drug dependence and no impact on patients' daily life. Therefore, in the field of chronic pain treatment, pulsed radiofrequency has significant safety advantages and is highly trusted.

## Conclusion

This review provides a fresh perspective and insight into the great potential of pulsed radiofrequency in the management of chronic pain. In retrospective studies, pulsed radiofrequency technology has won wide recognition for its unique efficacy and safety.

For chronic cervicogenic pain and chronic joint pain, pulsed radiofrequency has accumulated sufficient evidence to establish its status as a highly effective treatment. Meanwhile, pulsed radiofrequency has also demonstrated excellent efficacy in the treatment of postherpetic neuralgia and chronic migraine. Despite PRF's prominence in the field of chronic pain management, its exact mechanism of action remains to be further explored. This uncertainty undoubtedly increases the difficulty for clinicians in predicting and judging its efficacy, and limits its further development in the field of pain management.

It is worth noting that the satisfaction of PRF in the treatment of chronic pain varies significantly between different diseases and patients. For example, satisfaction with PRF is generally higher in the treatment of cervicogenic headache and small joint pathology, whereas its efficacy is relatively limited in the treatment of other types of chronic pain. This variability in efficacy undoubtedly adds to the complexity of clinical decision making. In addition, the selection of indications for PRF is equally challenging. Although PRF has been shown to be effective in a variety of chronic pain treatments, the variability of its efficacy across indications cannot be ignored. Therefore, how to accurately select indications to improve the therapeutic efficacy of PRF and patient satisfaction will be a key issue for clinicians and researchers to tackle in the future.
